# Haplotype of the Lipoprotein(a) Gene Variants rs10455872 and rs3798220 Is Associated with Parameters of Coagulation, Fibrinolysis, and Inflammation in Patients after Myocardial Infarction and Highly Elevated Lipoprotein(a) Values

**DOI:** 10.3390/ijms25020736

**Published:** 2024-01-06

**Authors:** Sabina Ugovšek, Andreja Rehberger Likozar, Tina Levstek, Katarina Trebušak Podkrajšek, Janja Zupan, Miran Šebeštjen

**Affiliations:** 1 Division of Internal Medicine, University Medical Centre Ljubljana, Zaloška cesta 7, 1000 Ljubljana, Slovenia; 2 Faculty of Medicine, University of Ljubljana, Vrazov trg 2, 1000 Ljubljana, Slovenia; 3 Department of Vascular Diseases, University Medical Centre Ljubljana, Zaloška cesta 7, 1000 Ljubljana, Slovenia; andreja.rehbergerlikozar@kclj.si; 4 Laboratory for Translational Medical Biochemistry, Institute of Biochemistry and Molecular Genetics, Faculty of Medicine, University of Ljubljana, Vrazov trg 2, 1000 Ljubljana, Slovenia; tina.levstek@mf.uni-lj.si (T.L.); katarina.trebusakpodkrajsek@mf.uni-lj.si (K.T.P.); 5 Clinical Institute for Special Laboratory Diagnostics, University Children’s Hospital, University Medical Centre Ljubljana, Vrazov trg 1, 1000 Ljubljana, Slovenia; 6 Faculty of Pharmacy, University of Ljubljana, Aškerčeva cesta 7, 1000 Ljubljana, Slovenia; janja.zupan@ffa.uni-lj.si; 7 Department of Cardiology, University Medical Centre Ljubljana, Zaloška cesta 7, 1000 Ljubljana, Slovenia

**Keywords:** inflammation, hemostasis, lipoprotein(a), rs10455872, rs3798220, KIV-2 repeats

## Abstract

Lipoprotein(a) (Lp(a)) is an independent risk factor for future coronary events. Variants rs10455872 and rs3798220 in the gene encoding Lp(a) are associated with an increased Lp(a) concentration and risk of coronary artery disease. We aimed to determine whether in high-risk coronary artery disease patients these two genetic variants and the kringle IV type 2 (KIV-2) repeats are associated with impairment of inflammatory and hemostatic parameters. Patients after myocardial infarction with elevated Lp(a) levels were included. Blood samples underwent biochemical and genetic analyses. In carriers of the AC haplotype, the concentrations of tumor necrosis factor (TNF)-α (4.46 vs. 3.91 ng/L, *p* = 0.046) and plasminogen activator inhibitor-1 (PAI-1) (*p* = 0.026) were significantly higher compared to non-carriers. The number of KIV-2 repeats was significantly associated with the concentration of high-sensitivity C-reactive protein (ρ = 0.251, *p* = 0.038) and overall fibrinolytic potential (r = −0.253, *p* = 0.038). In our patients, a direct association between the AC haplotype and both TNF-α and PAI-1 levels was observed. Our study shows that the number of KIV-2 repeats not only affects proatherosclerotic and proinflammatory effects of Lp(a) but is also associated with its antifibrinolytic properties.

## 1. Introduction

Cardiovascular diseases remain a leading cause of morbidity and mortality in the developed world [1]. Yearly, more than 3 million people worldwide and approximately 300 per 100,000 individuals older than 18 years in Slovenia suffer from acute myocardial infarction [1,2]. Lipoprotein(a) (Lp(a)) has been recognized as a risk factor for future coronary events, independent of low-density lipoprotein cholesterol (LDL-C) concentration [3]. The concentration of Lp(a) varies between individuals up to 1000-fold, and the largest part of this variation was attributed to genetic modifications of the lipoprotein(a) gene, *LPA* [4]. The rs10455872 (intronic variant A > G with 7% frequency for the risk allele G) and rs3798220 (missense variant T > C with 2% frequency for the risk allele C) in the *LPA* explain 36% of the variation in Lp(a) levels [5]. Both variants were shown to be associated with increased Lp(a) values as well as with the incidence of coronary disease [5]. The carriers of one of these two variants possess a 1.5-fold higher risk of coronary disease than the non-carriers. [5]. Furthermore, in a meta-analysis by Wei et al., rs10455872 was found to be associated with coronary disease independently of statin-induced change in LDL-C and persisted in patients with LDL-C below 70 mg/dL [6]. In the process of atherosclerosis, Lp(a) not only is important as a proatherogenic risk factor but also plays a role in the inflammatory process and increases the vulnerability of the atherosclerotic plaque and its tendency to rupture. At the same time, Lp(a) escalates the thrombogenic potential and thereby amplifies the possibility of clot formation in the ruptured atherosclerotic bed and risk of an acute cardiovascular event [7].

The atherogenic properties of Lp(a) are due to the LDL-like particle consisting of apolipoprotein B-100 (apoB-100), which is similar to LDL-C [8]. However, Lp(a) is more prone to oxidation than LDL-C, which increases the uptake of Lp(a) into macrophages via scavenger receptors and thus the formation of foam cells, which mark the beginning of the atherosclerotic process [9]. The main components responsible for the pro-inflammatory properties of Lp(a) are oxidized phospholipids (ox-PLs) [10]. Ox-PLs are mainly bound to lipoproteins containing apolipoprotein (a) (apo(a)). Compared to LDL-C, the binding of ox-PLs to Lp(a) is significantly higher, and at the same time, the transition of ox-PLs from oxidized LDL-C (ox-LDL) to Lp(a) is possible, but not in the reverse direction [7]. Many studies have shown that the ox-PL/apoB ratio is a predictive factor for both the progression of atherosclerosis [11] and future cardiovascular events [12], independent of other risk factors except Lp(a). The rationale behind this is a strong positive association between ox-PL/apoB and Lp(a), suggesting that the proatherogenic properties of Lp(a) are due to ox-PLs bound to apo(a) [13]. Monocytes in the blood of patients with increased Lp(a) values express significantly more adhesion molecules on their surface than monocytes of patients with normal Lp(a) values [14]. Long-term exposure of mouse bone marrow cells to high concentrations of Lp(a) significantly increased the number of pro-inflammatory monocytes and macrophages. However, all these effects disappeared upon the addition of antibodies against ox-PLs [15].

In patients with increased Lp(a) values, the balance between coagulation and fibrinolysis is disrupted in favor of coagulation. Apo(a) shares more than 80% of its structure with plasminogen, and due to this structural similarity, Lp(a) binds to the receptor sites for plasminogen, and thereby prevents its fibrinolytic action [16]. The most important determinant of the size of apo(a) isoforms and thus the concentration and also the atherogenic properties of Lp(a) is the number of kringle IV-type 2 repeats [17]. In apparently healthy individuals, the concentration of Lp(a) in carriers of the minor allele of rs10455872 or rs3798220 was significantly higher than in non-carriers [18]. However, there were no differences between the groups in plasminogen concentration and fibrinolytic activity measured by euglobulin clot lysis time (ECLT) [18]. In in vitro conditions, the addition of plasma from patients carrying a minor allele of rs3798220 accelerated the coagulation of plasma clots and slowed their fibrinolysis [19]. Both genetic variants were determined in our previous study, where we found that the genotypes and haplotypes of rs10455872 and rs3798220 in *LPA* were associated with the concentration of Lp(a) but not with the functional and morphological properties of the arterial vessel wall in patients in the stable phase after myocardial infarction [20]. Despite receiving the optimal therapy and maximum tolerated dose of statins and, if necessary, ezetimibe, these patients possessed an LDL-C concentration above the recommended level [21], and all of them also had significantly increased Lp(a) values.

Here, we used the same patient cohort [20] to determine if the two single nucleotide polymorphisms (SNPs) in *LPA*, namely rs10455872 and rs3798220, and kringle IV type 2 (KIV-2) repeats are associated with impaired inflammatory parameters and parameters associated with disturbance in the coagulation–fibrinolytic balance.

## 2. Results

### 2.1. Patients’ Characteristics

We used the same patient cohort as in our previous study [20,22]. Briefly, 69 patients who had suffered myocardial infarction before the age of 55 years were studied. Their genotype and haplotype frequencies for the two SNPs were published previously [20]. Briefly, the frequency distributions for genotypes were as follows: 40 (AA), 25 (AG), and 5 (GG) patients for rs10455872, and 48 (TT), 21 (TC), and 1 (CC) patients for rs3798220. The haplotypes were defined as A > G for rs10455872 and T > C for rs3798220, and their frequencies were as follows: AT (58.7%), AC (16.3%), GT (24.9%), and GC (0.09%) [20]. Due to low frequency, the latter haplotype was excluded from the analysis. The frequency distributions were as follows: no AT (8 patients), one AT (42 patients), and two AT alleles (20 patients) for the AT haplotype, and no AC (48 patients), one AC (21 patients) and two AC alleles (1 patient) for the AC haplotype, and no GT (40 patients), one GT (25 patients), and two GT alleles (5 patients) for the GT haplotype. All patients received optimal drug therapy, including a statin at the maximum tolerated dose and, if necessary, ezetimibe. At the same time, all risk factors except LDL-C and Lp(a) were well regulated. Clinical characteristics and values of the biochemical parameters are shown in Table 1. There were no differences in basic clinical characteristics such as age, body mass index, blood pressure, and lipid profile between the genotype and haplotype subgroups in the previous study [20].

### 2.2. Associations between LPA Genotypes, Haplotypes, and Inflammatory Parameters

**Figure 1:**
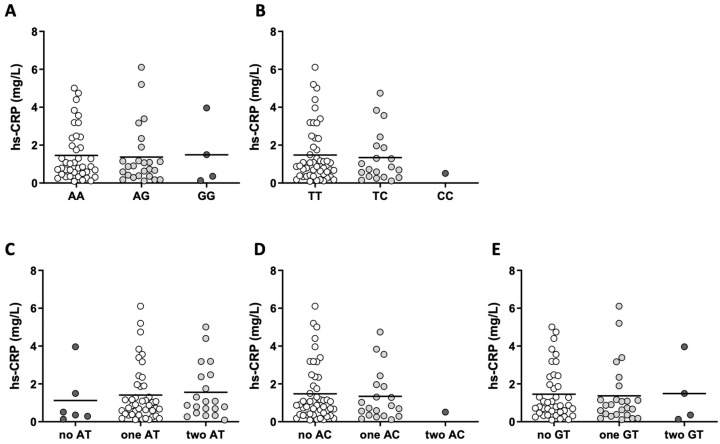
Levels of high-sensitivity C-reactive protein (hs-CRP) in all genotype (**A**,**B**) and haplotype (**C**–**E**) subgroups. Differences between the genotype and haplotype subgroups showed no statistical significance for hs-CRP (*p* > 0.05). Kruskal–Wallis (**A**,**C**,**E**) or Mann–Whitney U (**B**,**D**) tests were used to compare the differences in hs-CRP.

**Figure 2:**
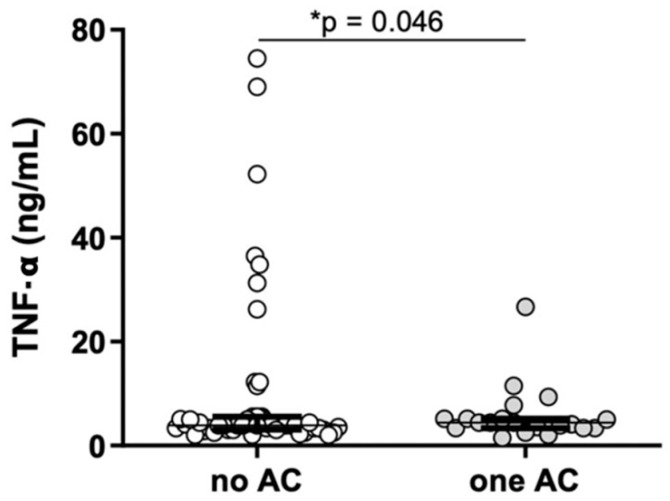
Tumor necrosis factor-α (TNF-α) concentrations in haplotype groups with one or no AC allele (Mann–Whitney U test, * *p* < 0.05).

Levels of hs-CRP levels in all genotype and haplotype subgroups are shown in Figure 1. The levels of other inflammatory parameters, namely, TNF-α, IL-6, IL-8, and VCAM-1, in all genotype and haplotype subgroups are given in Table 2. The concentration of TNF-α (Figure 2) was significantly higher (*p* = 0.046) in carriers of one AC haplotype (4.46 (3.36–5.14) ng/L) compared to non-carriers (3.91 (3.13–5.57) ng/L).

### 2.3. Associations between LPA Genotypes, Haplotypes, and Hemostatic Parameters

**Figure 3:**
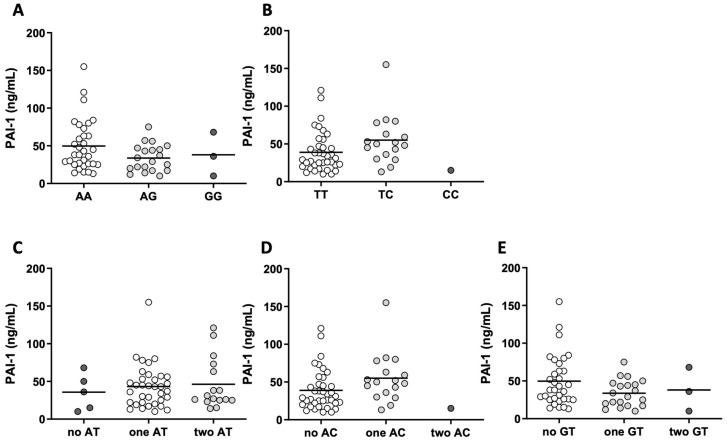
Plasminogen activator inhibitor-1 (PAI-1) levels in all genotype (**A**,**B**) and haplotype (**C**–**E**) subgroups. Kruskal–Wallis (**A**,**C**,**E**) or Mann–Whitney U (**B**,**D**) tests were used to compare the differences in PAI-1.

The levels of the hemostatic parameters, namely, PAI-1, TAFI, CHP, CFP, and OHP, in all genotype and haplotype subgroups are presented in Figure 3 and Table 3. In the TC genotype and in one AC allele carrier, PAI-1 concentration was significantly higher (*p* = 0.026 for both) compared to those with the TT genotype or without AC allele (Figure 3B,D). The remaining parameters of coagulation and fibrinolysis were not significantly different between the studied genotypes and haplotypes (Figure 3).

### 2.4. Associations between Lp(a) Levels, KIV-2 Repeats, and Inflammatory and Hemostatic Parameters

**Figure 4:**
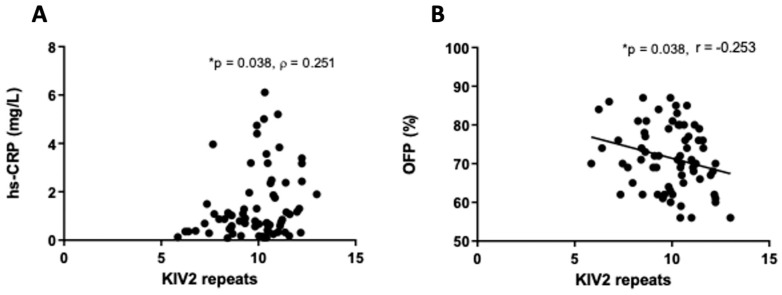
Correlation between inflammatory (**A**) and hemostatic markers (**B**) and the number of kringle IV type 2 (KIV-2) repeats. Pearson correlation (**A**) or Spearman correlation (**B**) analysis was used (* *p* < 0.05). hs-CRP, high-sensitivity C-reactive protein; OFP, overall fibrinolytic potential.

**Figure 5:**
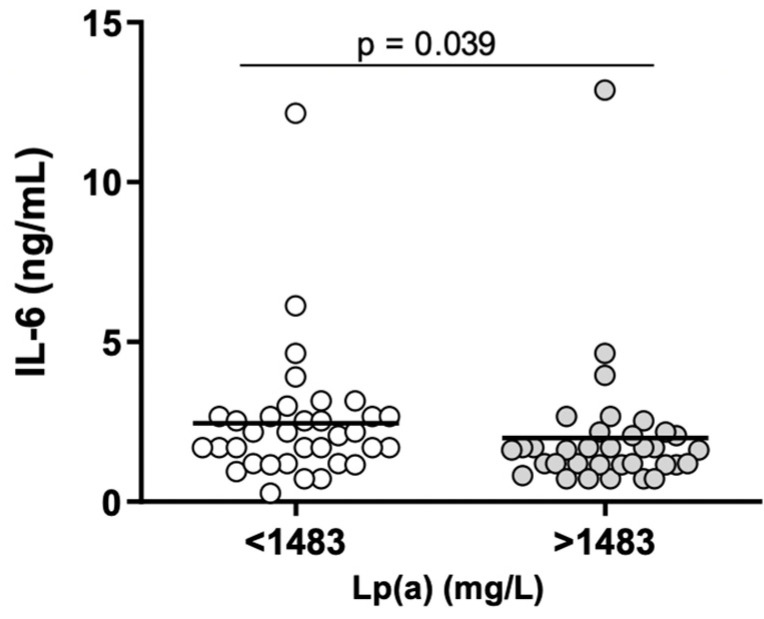
Interleukin (IL)-6 levels in two groups of lipoprotein(a) (Lp(a)), below and above Lp(a) median concentration, i.e., 1483 mg/L (Mann–Whitney U test, *p* = 0.039).

**Figure 6:**
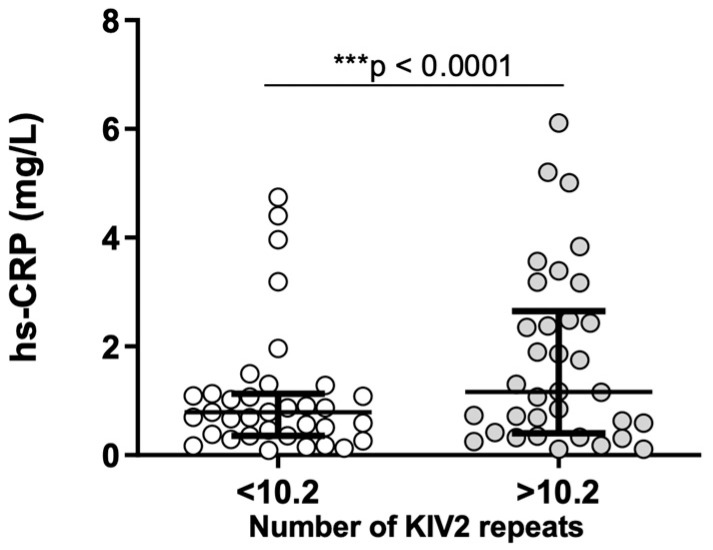
High-sensitivity C-reactive protein (hs-CRP) in two groups of number of kringle IV type 2 (KIV-2) repeats, below and above the median of KIV-2 repeats, i.e., 10.2 (Mann–Whitney U test, *** *p* < 0.0001).

**Figure 7:**
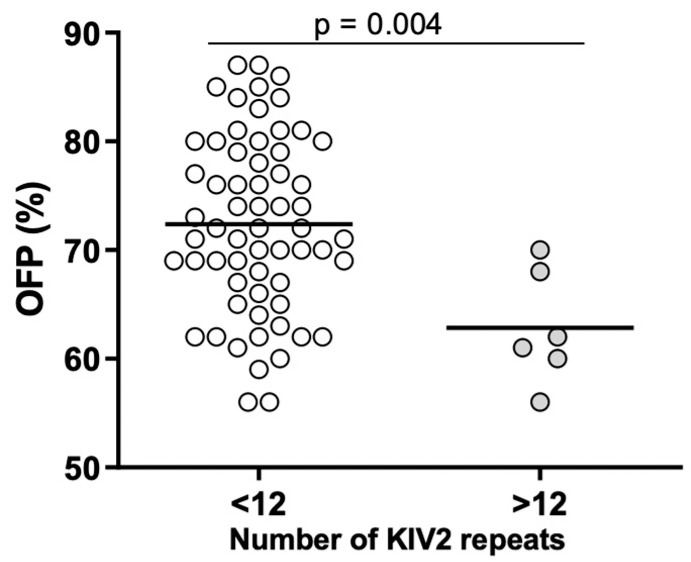
Overall fibrinolytic potential (OFP) in two groups of number of kringle IV type 2 (KIV-2) repeats, below and above 12 (Student’s *t* test, *p* < 0.01).

No correlations were found between Lp(a) concentration and parameters of inflammation and hemostasis. However, the number of KIV-2 repeats was significantly associated with the concentration of hs-CRP (ρ = 0.250; *p* = 0.038) and OFP (r = −0.253; *p* = 0.038) (Figure 4). Patients with Lp(a) concentration above the median value of 1483 mg/L had significantly lower (*p* = 0.039) IL-6 values (Figure 5). There were statistically significant differences (*p* = 0.01) between patients with number of KIV-2 repeats below or above the median value (10.2) for hs-CRP (Figure 6). The patients with KIV-2 repeats above the median had statistically higher hs-CRP values (1.17 (0.40–2.65)) compared to patients with KIV-2 repeats below the median (0.79 (0.36–1.13)). However, patients with number of KIV-2 repeats above 12 had a significantly higher OFP (*p* = 0.004) (Figure 7).

## 3. Discussion

The current study is the first to identify the influence of two previously well-recognized *LPA* variants on the parameters of inflammation, coagulation, and fibrinolysis in high-risk CAD patients. These two SNPs (rs10455872 and rs3798220) were previously associated with increased Lp(a) concentration and a risk of future cardiovascular events [13,23]. Furthermore, rs10455872 was associated with coronary disease independent of LDL-C levels [6]. Here, we measured the parameters of inflammation, coagulation, and fibrinolysis in our specific cohort of patients who had suffered a myocardial infarction before the age of 55. All of them were receiving antiplatelet drugs, beta blockers, ACE inhibitors, or sartans and statins at the maximum tolerated dose and ezetimibe as needed. Despite the optimal therapy, their LDL-C values were still not sufficiently controlled and all these patients had significantly increased Lp(a) values. In comparison with previous studies such as Clarke et al. [13] and Kamstrup et al. [23], where apparently healthy patients or patients after myocardial infarction were included regardless of Lp(a) values, we have included only patients after myocardial infarction with Lp(a) values higher than 600 mg/L. The number of KIV-2 repeats was significantly lower in our study, the relative value representing the average value of repeats on both alleles ranging from 6 to 15, whereas in Clarke et al. [13] and Kamstrup et al. [23], these numbers ranged from 6 to 99. However, these data are not directly comparable as different methods of determining the number of KIV-2 repeats were used. Interestingly, all studies [13,23], including ours [20], found the number of KIV-2 repeats to be statistically significantly associated with Lp(a) concentration.

The association between Lp(a) and inflammation is well known, and ox-PLs, one of the building blocks of Lp(a), are responsible for its pro-inflammatory action [10]. Arai et al. showed that in patients carrying the rs3798220 variants (CT or CC), both Lp(a) concentration and the ox-PL/apoB ratio were increased compared with non-carriers (TT), reflecting mainly ox-PLs on Lp(a) [24]. In their study, the concentration of ox-PLs was higher in patients with a number of KIV-2 repeats greater than 21, and at the same time, the concentration of Lp(a) was also higher in this group than in the group with a number of KIV-2 repeats less than 21. Ox-PL/apoB levels were associated with cardiovascular disease extent identified by coronary angiography [25], and with the extent and 5-year progression of carotid and femoral atherosclerosis independently of other cardiovascular risk factors except Lp(a) [11]. Despite very clear pathophysiological links between Lp(a) and inflammation, the clinical research in this area is scarce. Ansari et al. [26] genotyped more than 300 patients with angiographically confirmed coronary disease for rs10455872. They found that carriers of the GG genotype had significantly higher TNF-α concentration than carriers of the GT or TT genotype. However, no differences were detected in the concentration of IL-6, IL-10, and IL-18, while hs-CRP values were not measured. Since Ansari et al. [26] provided no data on Lp(a) values and treatment with drugs that affect both Lp(a) values and inflammatory parameters, in particular statins and acetylsalicylic acid, we cannot conclude whether this is a direct effect of rs10455872 or an indirect effect via Lp(a) concentration. In our study, the concentration of TNF-α was significantly lower in carriers of one allele of the AC haplotype compared to non-carriers. Given that we found no differences in Lp(a) concentration between these groups [20], we suggest that there is a direct association between the AC haplotype and TNF-α levels. We would like to emphasize that all our patients were treated with statins, which, regardless of their type and dose, increase the level of Lp(a) to the same extent [27]. Hence, the statin treatment could not have influenced this finding. The number of KIV-2 repeats, which is associated with both the Lp(a) concentration and the risk of future coronary events [23], was associated with an increased concentration of hs-CRP in our study. In the same cohort of patients, we previously found that polymorphisms in genes encoding CRP (rs1800947), TNF-α (rs1800629), and IL-6 (rs1800795) had no effect on the concentration of the corresponding inflammatory indicators [22]. Our previous study also showed that rs10455872 and rs3798220 were associated with higher Lp(a) concentration [20]. This is consistent with findings that the connection between Lp(a) and inflammation is mutual, as some inflammatory parameters, including IL-6 and TNF-α, increase the expression of *LPA* and thus its concentration [28]. On the other hand, oxPLs, as a component of Lp(a), stimulate the production of the same pro-inflammatory cytokines. The amount and activity of oxPLs are related to the size of apo(a) isoforms, which are associated with both rs10455872 and rs3798220 studied in our research [24].

Impaired fibrinolytic activity has been shown to be an independent predictor of myocardial infarction, both in apparently healthy subjects [29] and in patients with known CAD [30]. However, unlike coagulation, we do not have a method that reliably measures fibrinolytic capacity [31]. Measurements of the concentrations and/or activity of individual components of the fibrinolytic system, such as PAI-1, TAFI, and D-dimer, do not satisfactorily demonstrate the functioning of the fibrinolytic system as a whole. Therefore, we also measured OHP in addition to the individual components of the fibrinolytic system. The OHP is an assay that measures the overall hemostatic potential and has been evaluated in connection with hypercoagulability in patients with CAD [32]. In addition to OHP, we can also measure OCP and calculate OFP, giving a more detailed insight into the functioning of the hemostatic system [33].

The antifibrinolytic potential of Lp(a) is due to the very similar structure of apo(a) and plasminogen. Apo(a) does not possess its own profibrinolytic activity but competes for the binding sites for plasminogen, thereby reducing the fibrinolytic efficiency of plasminogen [34]. There are slightly more studies on the influence of rs3798220 and rs10455872 on hemostatic parameters compared to studies on the influence of these two SNPs on inflammation. However, the data are still scarce. Scipione et al. [19] found that the isoleucine-to-methionine substitution (encoded by rs3798220) accelerates the coagulation of plasma clots in vitro and inhibits lysis to a greater extent than the wild-type variant. Given that Scipione et al. [19] also found that the increased concentration of Lp(a) itself contributes to the antifibrinolytic activity, as does the previously mentioned variant, it could be concluded that in addition to the increased concentration of Lp(a), the structure of apo(a) is also important as it further increases the antifibrinolytic activity and cardiovascular risk. However, we have to keep in mind that this is an in vitro study. In our study, PAI-1 concentration was significantly higher in carriers of one TC genotype and one allele of the AC haplotype compared to the TT genotype or non-carriers of the AC allele. Given that endothelial cells are one of the most important sites of PAI-1 production, it might be that endothelial cells are more activated in the TC genotype or one allele of the AC haplotype carriers despite the same atherogenic lipoprotein values between the patients. Further functional studies in cell models are needed to confirm this. Even more important is our finding that OFP was related to the number of KIV-2 repeats. The number of KIV-2 repeats not only is related to Lp(a) concentration, as we also showed in our research, but also determines the structure of apo(a) [35]. The number of KIV-2 repeats thus affects not only the concentration of Lp(a), and thus its proatherosclerotic and proinflammatory effects, but also the structure of apo(a) and its associated antifibrinolytic properties. However, the antifibrinolytic activity may also depend on the ratio between the concentration of plasminogen and apo(a), which was evident for carriers of both rs10455872 and rs3798220 variants [18]. Given that smaller apo(a) isoforms have a greater affinity for binding to fibrin than larger ones [36], we could assume that in addition to the ratio between total apo(a) or Lp(a) and fibrinogen, the ratio between the smaller apo(a) isoforms and plasminogen plays a particularly important role. Different apo(a) isoforms have different antifibrinolytic effects that depend on their affinity for binding to fibrin. With greater affinity for binding to fibrin, smaller isoforms of apo(a) have the same or even lower enzymatic activity. As more apo(a) or Lp(a) is bound to fibrin instead of fibrinogen, the fibrinolytic activity is consequently reduced.

## 4. Patients and Methods

### 4.1. Patients

The same patient cohort was used as in our previous studies [20,22]. Briefly, 69 patients aged between 18 and 65 years with clinically stable coronary artery disease (CAD) at least 6 months after myocardial infarction were included in this study. Participants with first acute coronary event before the age of 55 and Lp(a) level above 1000 mg/L or an Lp(a) level above 600 mg/L and an LDL-C level of more than 2.6 mmol/L were eligible. All patients received statins at the highest tolerated dose, along with ezetimibe where needed, as well as beta blockers, angiotensin-converting enzyme inhibitors/angiotensin II receptor blockers, and antiplatelet drugs. The exclusion criteria were elevated liver transaminases, by more than three times above the normal levels, severe renal impairment and serum creatinine >200 mmol/L, or history of acute illness in the previous 6 weeks. All the procedures performed in this study that involved human participants were carried out in accordance with the ethical guidelines of the 1964 Declaration of Helsinki. Approval for this study was obtained from the National Medical Ethics Committee of the Republic of Slovenia (reference number: KME 0120-357/2018/8). This study is registered with CinicalTrials under the number NCT04613167. All patients signed a written informed consent form prior to inclusion in this study.

### 4.2. Biochemical Analysis

Blood for laboratory analysis was collected and biochemical analysis performed as described previously [20]. The blood for laboratory analysis was drawn in the morning after 12 h of fasting. Samples were collected from the antecubital vein into vacuum-sealed 5 mL tubes containing clot activator (Vacutube, LT Burnik, Slovenia). Serum was obtained by 15 min centrifugation at 2000× *g*. Analysis of total cholesterol (TC), triglycerides (TG), high-density lipoprotein cholesterol (HDL-C), apolipoprotein A1 (apoA1), and apolipoprotein B100 (apoB) was performed by standard colorimetric or immunologic assays. The Friedewald formula [37] was used to calculate LDL-C. Thrombin-activatable fibrinolysis inhibitor (TAFI) activity in plasma was measured by using the chromogenic method utilizing Pefakit^®^ TAFI reagents (Pentapharm, Basel, Switzerland) on an automated coagulation analyzer CS-2500 (Sysmex, Kobe, Hyogo, Japan). Plasminogen activator inhibitor-1 (PAI-1) antigen in plasma was measured using a classic sandwich ELISA (ASSERACHROM PAI-1, Diagnostica Stago, Asnières-Sur-Seine, France). Measurements of tumor necrosis factor-α (TNF-α), high-sensitivity C-reactive protein (hs-CRP), interleukin (IL)-6, IL-8, and IL-10 levels in serum were measured by using the Luminex’s xMAP^®^ Technology utilizing magnetic beads coupled with specific antibodies, which allowed multiplexing. All analyses were performed according to the manufacturer’s instructions (R&D Systems, Minneapolis, MN, USA). Overall coagulation potential (OCP), overall hemostatic potential (OHP), and overall fibrinolytic potential (OFP) were measured and calculated as described previously [33].

### 4.3. Genetic Analysis

Genetic analyses were performed at the Institute for Biochemistry and Molecular Genetics, Faculty of Medicine, University of Ljubljana as previously described [20,22]. Briefly, genomic DNA was extracted from venous blood samples by using FlexiGene DNA kit 250 (Qiagen, Hilden, Germany). The *LPA* genotyping was performed by using Predesigned TaqMan SNP genotyping assays (C_30016089_10, C_25930271_10; Applied Biosystems, Waltham, MA, USA) and TaqMan™ Genotyping Master Mix (Applied Biosystems, Waltham, MA, USA) according to the manufacturer’s instructions. Analysis of *LPA* kringle repeats was performed by using custom TaqMan expression assay for exon 5 of the *LPA* gene. In brief, genomic DNA was diluted to 10 ng/µL with nuclease-free water. The PCR reaction mix contained 5 µL TaqMan Universal PCR Master Mix, 0.25 µL TaqMan Genotyping Assay Mix (40×), and 2.75 µL DNase-free water, to which we added 2 µL of the pre-diluted DNA. Genotyping PCR was performed with a QuantStudio 7 Flex Real-Time PCR System (Applied Biosystems, Waltham, MA, USA).

### 4.4. Statistical Analysis

Kolmogorov–Smirnov tests were used to define variables showing normal distributions, with these data expressed as mean values and standard deviations. The non-normally distributed variables are expressed as median values and range (lower and upper quartiles). To calculate the differences between the three subgroups of genotypes or haplotypes, one-way ANOVA was used for variables showing normal distributions, and Kruskal–Wallis test for non-normally distributed variables. The group with a single patient (carrier of the CC genotype and AC haplotype) was not included in the statistical analysis. Hence, to calculate the differences between the two groups of rs3798220 genotypes (TT vs. CT, Figure 1B and Figure 3B) and AC haplotypes (one AC vs. no AC allele, Figure 1D, Figure 2, and Figure 3D), Student’s *t* tests were used for variables showing normal distributions and Mann–Whitney U tests for non-normally distributed variables. Pearson or Spearman correlation analysis was performed to determine the correlation of KIV-2 repeats with biochemical parameters. Statistical analysis was performed by using IBM SPSS Statistics for Windows (IBM Corp., version 25.0, Armonk, NY, USA) and GraphPad Prism version 8 for Windows (GraphPad Software, San Diego, CA, USA). G Power was used to perform the power calculations [38]. *p* values < 0.05 were considered statistically significant.

## 5. Conclusions

Our research is the first to study the influence of two *LPA* variants, rs3798220 and rs10455872, on the parameters of inflammation and hemostasis. The selection of the patients in our study has both an advantage and a shortcoming. The shortcoming is the inclusion of a relatively small number of patients. This is due to strict inclusion criteria and the dropout of patients and samples during this study. The advantage is that all our patients had significantly elevated Lp(a) values, which on one hand allowed us to treat significantly high-risk patients. However, on the other hand, we were not able to show the association of Lp(a) values and thus also the number of KIV-2 repeats with the parameters of inflammation and hemostasis on a wider population. In addition, we have to take into account that all our patients received statins at the highest tolerated doses. Statins increase the concentration of lipoprotein(a) more in people with smaller apo(a), i.e., less KIV-2 recurrence [39]. On the other hand, statins have a beneficial effect on the parameters of coagulation, fibrinolysis, and inflammation [40]. While we cannot expect a group of post-myocardial infarction patients to have similar characteristics, some answers could be provided by a group of patients treated with non-specific Lp(a)-lowering drugs that are not currently in clinical use. Carriers of one allele of the AC haplotype in *LPA* were shown to have higher concentrations of both TNF-α and PAI-1. This could indicate that these patients are prone to higher risk due to more severe inflammation and impaired fibrinolytic activity. The number of KIV-2 repeats, which in addition to Lp(a) concentration also affects the size of apo(a) isoforms, was associated with OFP. To determine the influence of genetic variants in *LPA* on the parameters of inflammation and hemostasis, further studies would have to include a larger number of patients with a wider spectrum of Lp(a) values receiving no therapy that affects Lp(a) or the parameters of inflammation and hemostasis.

## Figures and Tables

**Table 1:** Clinical and biochemical characteristics of the patients.

Parameter	Value (*n* = 69)
Age (years)	51.7 ± 8.7
Body mass index (kg/m^2^)	28.7 ± 8.7
Systolic blood pressure (mmHg)	128 ± 15
Diastolic blood pressure (mmHg)	77 ± 9
Total cholesterol (mmol/L)	4.24 ± 0.83
HDL cholesterol (mmol/L)	1.20 ± 0.28
LDL cholesterol (mmol/L)	2.35 ± 0.70
Triglycerides (mmol/L)	1.65 ± 0.79
Lipoprotein(a) (mg/L)	1431 (11207–1783)
Apolipoprotein B (g/L)	0.81 ± 0.22
Apolipoprotein A1 (g/L)	1.34 ± 0.19
KIV-2 repeats (*n*)	10.2 (5.8–13.5)
hs-CRP (mg/L)	0.87 (0.37–1.93)
TNF-α (ng/L)	3.91 (3.36–5.36)
IL-6 (ng/L)	1.69 (1.20–2.53)
IL-8 (ng/L)	13.50 (10.65–18.35)
D-dimer (µg/L)	270 (190–482)
F VIII (IU/mL)	1.56 (1.24–1.74)
TAFI (%)	100.50 (86.00–110.25)
PAI-1 (ng/mL)	36.50 (22.25–56.75)
OCP (Abs-sum)	23.45 (19.78–28.13)
OHP (Abs-sum)	6.60 (4.73–8.65)
OFP (%)	71.54 ± 8.32

Data are median (lower–upper quartiles) or mean values ± standard deviation. HDL, high-density lipoprotein; LDL, low-density lipoprotein; KIV-2, kringle IV type 2; hs-CRP, high-sensitivity C-reactive protein; TNF-α, tumor necrosis factor-α; IL, interleukin; F VIII, factor VIII; TAFI, thrombin-activatable fibrinolysis inhibitor; PAI-1, plasminogen activator inhibitor-1; OCP, overall coagulation potential; OHP, overall hemostatic potential; OFP, overall fibrinolytic potential.

**Table 2:** Inflammatory parameters for the different *LPA* genotype and haplotype subgroups.

Parameter	Subgroup			*p* Value
**rs10455872**	**A/A (*n* = 40)**	**A/G (*n* = 25)**	**G/G (*n* = 5)**	
IL-6 (ng/L)	1.69 (1.20–2.44)	2.07 (1.41–2.67)	2.07 (1.41–2.67)	0.328
IL-8 (ngl/L)	12.55 (10.43–17.30)	14.60 (11.40–36.65)	2.56 (0.83–5.59)	0.383
TNF-α (ng/L)	3.91 (3.36–5.14)	3.91 (3.21–16.12)	4.56 (3.31–5.10)	0.921
VCAM-1 (ng/L)	0.62(0.50–0.89)	0.65 (0.54–0.93)	0.52 (0.29–1.43)	0.489
**rs3798220**	**T/T (*n* = 48)**	**T/C (*n* = 21)**	**C/C (*n* = 1)**	
IL-6 (ng/L)	1.69 (1.17–2.67)	1.66 (1.20–2.15)	2.18	0.558
IL-8 (ng/L)	13.00 (10.50–17.48)	16.30 (10.70–20.50)	17.90	0.524
TNF-α (ng/L)	3.91 (3.13–5.57)	4.46 (3.36–5.14)	3.91	0.940
VCAM-1 (ng/L)	0.60 (0.51–0.86)	0.78 (0.51–0.92)	0.72	0.445
**Haplotype AT**	**no AT (*n* = 8)**	**one AT (*n* = 42)**	**two AT (*n* = 20)**	
IL-6 (ng/L)	1.90 (1.05–4.50)	1.69 (1.20–2.67)	1.45 (1.17–2.53)	0.640
IL-8 (ng/L)	17.65 (6.90–34.80)	14.60 (11.30–20.60)	10.90 (10.18–15.48)	0.281
TNF-α (ng/L)	4.56 (3.70–10.52)	3.91 (3.36–5.14)	3.91 (3.13–10.03)	0.790
VCAM-1 (ng/L)	0.61 (0.40–0.97)	0.78 (0.53–0.92)	0.59 (0.47–0.64)	0.087
**Haplotype AC**	**no AC (*n* = 48)**	**one AC (*n* = 21)**	**two AC (*n* = 1)**	
IL-6 (ng/L)	1.69 (1.17–2.67)	1.66 (1.20–2.15)	2.18	0.558
IL-8 (ng/L)	13.00 (10.50–17.48)	16.30 (10.70–20.50)	17.90	0.524
TNF-α (ng/L)	3.91 (3.13–5.57)	4.46 (3.36–5.14)	3.91	0.940
VCAM-1 (ng/L)	0.60 (0.51–0.86)	0.78 (0.51–0.92)	0.72	0.445
**Haplotype GT**	**no GT (*n* = 40)**	**one GT (*n* = 25)**	**two GT (*n* = 5)**	
IL-6 (ng/L)	1.69 (1.20–2.44)	2.07 (1.41–2.67)	2.56 (0.83–5.59)	0.328
IL-8 (ng/L)	12.55 (10.43–17.30)	14.60 (11.40–36.65)	13.05 (3.30–19.95)	0.383
TNF-α (ng/L)	3.91 (3.36–5.14)	3.91 (3.21–16.12)	4.56 (3.31–5.10)	0.921
VCAM-1 (ng/L)	0.62 (0.50–0.89)	0.65 (0.54–0.93)	0.52 (0.29–1.43)	0.489

Data are median (lower–upper quartiles) or mean values ± standard deviation except for C/C and two alleles of the AC haplotype, where only one subject was genotyped. *p* values for rs3798220 and the AC haplotype were obtained using Student’s *t* tests for variables shown as mean values ± standard deviation, and Mann–Whitney U tests for variables shown as median values (lower–upper quartiles). Other *p* values were obtained using one-way ANOVA for variables presented as mean values ± standard deviation, and Kruskal–Wallis test for variables shown as median values (lower–upper quartiles). IL, interleukin; TNF-α, tumor necrosis factor-α; VCAM-1, vascular cell adhesion molecule 1.

**Table 3:** Hemostatic parameters for the different *LPA* genotype and haplotype subgroups.

Parameter	Subgroup			*p* Value
**rs10455872**	**A/A (*n* = 40)**	**A/G (*n* = 25)**	**G/G (*n* = 4)**	
D-dimer (µg/L)	270.00 (190.00–550.00)	268.00 (190.00–385.75)	313.00 (203.75–429.00)	0.786
TAFI (%)	102.00 (88.25–110.25)	93.00 (86.50–110.25)	93.50 (76.00–122.25)	0.784
OCP (Abs-sum)	24.45 (20.03–29.60)	23.10 (19.55–27.10)	23.50 (16.80–24.60)	0.715
OHP (Abs-sum)	6.50 (4.65–8.63)	6.70 (4.70–8.60)	6.00 (5.00–7.00)	0.946
OFP (%)	72.00 ± 8.65	71.16 ± 8.18	68.67 ± 6.11	0.819
**rs3798220**	**T/T (*n* = 48)**	**T/C (*n* = 20)**	**C/C (*n* = 1)**	
D-dimer (µg/L)	270.00 (190.00–421.00)	295.00 (192.00–649.00)	190.00	0.299
TAFI (%)	100.00 (85.00–111.00)	100.00 (89.75–109.25)	113.00	0.519
OCP (Abs-sum)	23.40 (19.60–27.80)	24.05 (19.78–30.20)	30.00	0.362
OHP (Abs-sum)	6.30 (4.70–8.30)	7.40 (5.53–8.90)	6.70	0.583
OFP (%)	71.68 ± 8.16	70.90 ± 8.95	78.00	0.615
**Haplotype AT**	**no AT (*n* = 6)**	**one AT (*n* = 43)**	**two AT (*n* = 20)**	
D-dimer (µg/L)	217.50 (190.00–397.00)	279.00 (192.00–526.00)	252.50 (190.00–484.50)	0.460
TAFI (%)	99.00 (78.00–116.50)	97.50 (88.25–109.75)	102.00 (84.25–112.50)	0.997
OCP (Abs-sum)	23.50 (19.35–31.05)	23.40 (19.60–28.20)	22.40 (20.20–27.73)	0.932
OHP (Abs-sum)	6.50 (5.50–9.50)	27.10 (4.70–8.70)	5.60 (4.45–8.15)	0.558
OFP (%)	70.80 ± 5.93	71.09 ± 8.63	72.70 ± 8.37	0.737
**Haplotype AC**	**no AC (*n* = 48)**	**one AC (*n* = 20)**	**two AC (*n* = 1)**	
D-dimer (µg/L)	270.00 (190.00–421.00)	295.00 (192.00–649.00)	190.00	0.299
TAFI (%)	100.00 (85.00–111.00)	100.00 (89.75–109.25)	113.00	0.519
OCP (Abs-sum)	23.40 (19.60–27.80)	24.05 (19.78–30.20)	30.00	0.362
OHP (Abs-sum)	6.30 (4.70–8.30)	7.40 (5.53–8.90)	6.70	0.583
OFP (%)	71.68 ± 8.16	70.90 ± 8.95	78.00	0.615
**Haplotype GT**	**no GT (*n* = 40)**	**one GT (*n* = 25)**	**two GT (*n* = 4)**	
D-dimer (µg/L)	270.00 (190.00–550.00)	268.00 (190.00–385.75)	313.00 (203.75–429.00)	0.786
TAFI (%)	102.00 (88.25–110.25)	93.00 (86.50–110.25)	93.50(76.00–122.25)	0.784
OCP (Abs-sum)	24.45 (20.03–29.60)	23.10 (19.55–27.10)	23.50 (16.80–27.40)	0.715
OHP (Abs-sum)	6.50 (4.65–8.63)	6.70 (4.70–8.60)	6.00 (5.00–7.00)	0.946
OFP (%)	72.00 ± 8.65	71.16 ± 8.18	68.67 ± 6.11	0.819

Data are median values (lower–upper quartiles) or mean values ± standard deviation except for C/C and two alleles of the AC haplotype, where only one subject was genotyped. *p* values for rs3798220 and the AC haplotype were obtained using Student’s *t* tests for variables shown as mean values ± standard deviation, and Mann–Whitney U tests for variables shown as median values (lower–upper quartiles). Other *p* values were obtained using one-way ANOVA for variables presented as mean values ± standard deviation, and Kruskal–Wallis test for variables shown as median values (lower–upper quartiles). TAFI, thrombin-activatable fibrinolysis inhibitor; OCP, overall coagulation potential; OHP, overall hemostatic potential; OFP, overall fibrinolytic potential.

## Data Availability

The data presented in this study are available on reasonable request from the corresponding author. The data are not publicly available due to patient data privacy.
